# Application of Pulsed Laser Deposition in the Preparation of a Promising MoS*_x_*/WSe_2_/C(В) Photocathode for Photo-Assisted Electrochemical Hydrogen Evolution

**DOI:** 10.3390/nano11061461

**Published:** 2021-05-31

**Authors:** Roman Romanov, Vyacheslav Fominski, Maxim Demin, Dmitry Fominski, Oxana Rubinkovskaya, Sergey Novikov, Valentin Volkov, Natalia Doroshina

**Affiliations:** 1 National Research Nuclear University MEPhI (Moscow Engineering Physics Institute), Kashirskoe sh., 31, 115409 Moscow, Russia; limpo2003@mail.ru (R.R.); dmitryfominski@gmail.com (D.F.); oxygenofunt@gmail.com (O.R.); 2 Immanuel Kant Baltic Federal University, A. Nevskogo St 14, 236016 Kaliningrad, Russia; sterlad@mail.ru; 3 Center for Photonics and 2D Materials, Moscow Institute of Physics and Technology (MIPT), 141700 Dolgoprudny, Russia; novikov.s@mipt.ru (S.N.); vsv.mipt@gmail.com (V.V.); doroshina.nv@phystech.edu (N.D.)

**Keywords:** hydrogen evolution, pulsed leaser deposition, heterostructure, photoelectrocatalysis, semiconductors

## Abstract

We studied the possibility of using pulsed laser deposition (PLD) for the formation of a MoS*_x_*/WSe2 heterostructure on a dielectric substrate. The heterostructure can be employed for effective solar water splitting to produce hydrogen. The sapphire substrate with the conducting C(B) film (rear contact) helped increase the formation temperature of the WSe_2_ film to obtain the film consisting of 2H-WSe_2_ near-perfect nanocrystals. The WSe_2_ film was obtained by off-axis PLD in Ar gas. The laser plume from a WSe_2_ target was directed along the substrate surface. The preferential scattering of selenium on Ar molecules contributed to the effective saturation of the WSe_2_ film with chalcogen. Nano-structural WSe_2_ film were coated by reactive PLD with a nanofilm of catalytically active amorphous MoS*_x_*_~4_. It was established that the mutual arrangement of energy bands in the WSe_2_ and MoS*_x_*_~4_ films facilitated the separation of electrons and holes at the interface and electrons moved to the catalytically active MoS*_x_*_~4_. The current density during light-assisted hydrogen evolution was above ~3 mA/cm^2^ (at zero potential), whilst the onset potential reached 400 mV under irradiation with an intensity of 100 mW/cm^2^ in an acidic solution. Factors that may affect the HER performance of MoS*_x_*_~4_/WSe_2_/C(В) structure are discussed.

## 1. Introduction

Transition metals chalcogenides have received considerable attention from scientists involved in the development of photoelectrochemical cells for producing hydrogen by solar water splitting [1,2,3]. These semiconducting materials have physio-chemical properties that enable their usage as both photo-active materials and hydrogen evolution electrocatalysts [4,5,6]. The good catalytic properties of metal chalcogenides (particularly amorphous molybdenum sulfides MoS*_x_*) allow the compounds to replace expensive platinum. Moreover, they can ensure high efficiency of photo-assisted hydrogen evolution when using silicon-based heterostructures (n+p-Si) [7,8,9]. The photoactivity of crystalline transition metal dichalcogenides is sufficient for creating photocathodes based on these materials. Efficient hydrogen evolution is usually achieved by using an expensive (Pt/Ru) cocatalyst [10,11].

It is essential to coalesce the useful semiconductive and catalytic properties of transition metal chalcogenides in creating hybrid or heterostructures. These structures consist entirely of thin-film metal chalcogenides that have been selected for their structure and chemical composition. These compounds also act as photo-assistant agents in water splitting for hydrogen production [12].

Tungsten diselenide is a promising photo-active transition metal dichalcogenide [12,13,14,15]. Crystalline WSe_2_ is a p-type semiconductor with a small band gap (~1.1 eV). If the conductivity is sufficiently high, this compound can be combined with catalytically active n-type metal chalcogenides to form photocathodes. The central requirement for WSe_2_ films used in such photocathodes arises from the need to obtain a nearly perfect structure with minimum defects, including edge states. The recombination rate of nonequilibrium carriers (electrons and holes), which form during irradiation, decreases in the process. However, nanostructured WSe_2_ films can have a greater area for hydrogen evolution, while edge states can be passivated by a co-catalyst [10,13]. WSe_2_ films with a nearly perfect crystal lattice are usually obtained by chemical synthesis (from the vapour phase or in a special solution) or by the selenization of thin-film precursors (for instance, [16,17]). These techniques have both advantages and disadvantages. Thus, finding alternative techniques to obtain thin WSe_2_ film with targeted properties remains a challenge.

In its traditional on-axis configuration, pulsed layer deposition (PLD) makes it possible to create WSe_2_ with a crystal structure and good catalytic properties [18,19,20,21]. However, when using the on-axis PLD to obtain WSe_2_ films, problems were revealed with obtaining a stoichiometric composition with a perfect chemical state of atoms in the film. This situation can be attributed to several factors, including the preferential sputtering of selenium on a growing WSe_2_ when exposed to laser-plasma and the propensity of selenium to form pure Se nanoparticles at room temperature or to be desorbed at higher substrate temperatures [20,22,23,24]. Submicron- and nanoscale particles of metal W can be introduced into the film [25]. These particles form upon laser irradiation of a WSe_2_ target.

In the case of on-axis PLD geometry, the substrate is placed normally to the axis of the laser plume expansion. Fominski et al. [26] have established that, under some on-axis PLD regimes, using a buffer gas makes it possible to decrease the efficiency of the preferential self-sputtering of chalcogen atoms. However, this technique suffers from considerable limitations: under some regimes of ablation of a dichalcogenide metal target, a laser plume may form and localize in a narrow solid angle. It has been revealed that chalcogen atoms can move to the plume periphery, while a buffer gas cannot preclude the metallization of the centre of the film deposition area [27].

Experiential studies of amorphous molybdenum sulfide (a-MoS*_x_*) catalytic film creation have shown that, during off-axis PLD, the buffer gas has the conditions necessary for the effective saturation of films with chalcogen atoms [28]. These conditions are a result of the difference between the S and Mo atomic masses. During off-axis PLD, the substrate is placed parallel to the plume expansion. The film grows chiefly through the deposition of atoms that have collided with buffer gas molecules and changed their direction. The same effect can be achieved during the off-axis PLD of WSe_2_ films due to the substantial difference between the W and Se atomic masses. One of the goals of this study was to test whether the off-axis PLD technique could be applied to a WSe_2_ target to obtain WSe_2_ nanocrystal films that are nearly perfect in terms of structure and chemical state.

Films based on a-MoS*_x_*-nanomaterial have a high electrocatalytic activity during hydrogen evolution reaction (HER) [7,13,29,30,31]. The most common technique to obtain such films is a chemical synthesis or chemical deposition in the solutions of special precursors. The applicability of a laser-based technique to create amorphous a-MoS*_x_* films with good electrocatalytic properties has been described in the literature [28,32,33,34,35]. Fominski et al. demonstrate that the PLD technique is associated with the highest catalytic activity in MoS*_x_*_~4_ films because they contain Mo_3_S_12_/Mo_3_S13 clusters [35]. It is suggested that reactive PLD (RPLD) in H_2_S gas be used to create thin homogenous а-MoS*_x_* films with a high content of catalytically active states of sulfur [34]. Performing on-axis RPLD from a Mo target prevents the formation of a substantial number of particles of various sizes during ablation. It also ensures a relatively conformal coating of a rough surface, which is typical of catalysts. This beneficial effect is possible because deposition is carried out using a flux of Mo atoms, which scatter at different angles once collided with H_2_S molecules. Given the difficulty of predicting the conductivity type in a-MoS*_x_* films with increased S content, it was necessary to establish whether the mutual arrangement of energy bands in the MoS*_x_*_~4_/WSe_2_ heterostructure is optimal for the effective separation of electron-hole pairs when light irradiated during the photo-assisted HER.

It is widely accepted that the material of the rear contact to the semiconductor can have a pronounced effect on the current transport in the semiconductor photovoltaic structure and probably also the photoelectrocatalyst [36]. We investigated boron-doped carbon films as rear contacts. A preliminary study showed that the introduction of boron atoms could produce p-type conductivity of C(B) films [37]. Films with good conductivity and mechanical strength were created by PLD from a mixed boron/graphite target. The substrate of the heterostructure was a sapphire plate. However, sapphire can be replaced by a cheaper material—glass, quartz, etc.

Our study aimed to form a multi-layered MoS*_x_*_~4_/WSe_2_/C(В) by PLD. The structure had to contain thin-film nanomaterials with properties sufficient for an effective photo-assisted HER in an acidic solution. When selecting PLD conditions for obtaining these nanomaterials, we used the results of a preliminary investigation of each nanomaterial. After the heterostructure have been assembled (i.e., after layer-by-layer nanomaterial deposition), chosen PLD conditions may prove to be non-optimal for efficient photoelectrocatalysis of hydrogen evolution. Nonetheless, the findings made it possible to produce recommendations on how laser-based processes may be improved and the structure and composition of selected nanomaterials modified.

## 2. Materials and Methods

### 2.1. Experimental Methods for On-Axis and Off-Axis PLD of Functional Nanolayers for MoS_x~4_/WSe_2_/C(В) Heterostructure Formation

**Figure 1:**
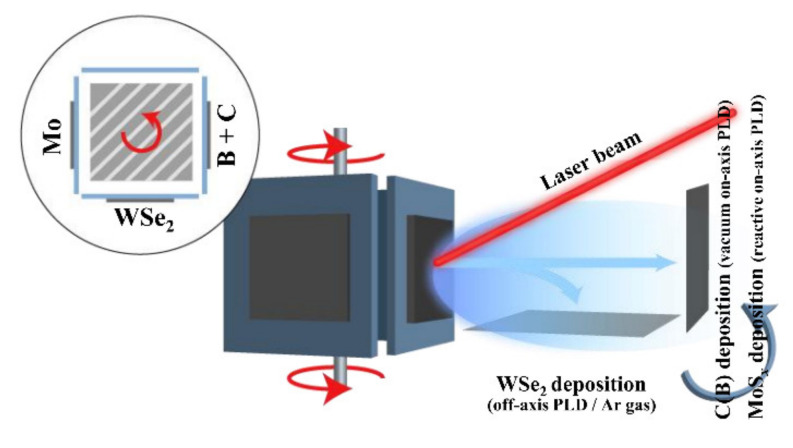
A schematic of the PLD technique employed to form functional layers in a MoS*_x_*_~4_/WSe_2_/C(В) heterostructure on a sapphire substrate. Comments are given in the text.

Figure 1 shows the mutual arrangement of the target and the substrate when using on-axis and off-axis PLD for the formation of a MoS*_x_*_~4_/WSe_2_/C(В) heterostructure. The on-axis PLD configuration was used to deposit a C(B) film. The target, which consisted of a carbon (soot) and boron powder mixture in the proportion С/В~6, was concurrently ablated. For more detail on target manufacturing and the selection of laser ablation, see [37]. A Solar LQ529 laser (Minsk, Belarus) was used to ablate the target. The pulse duration and energy were 10 ns and 100 mJ respectively. The pulse repetition rate was 20 Hz. The energy density on the surface of the C(B) target was 9 J/cm^2^. The substrate was placed to the laser plume axis, 3 cm away from the target, and heated to 500 °С. The deposition was performed in a vacuum at a residual pressure of 5 × 10^−4^ Pa. The deposition period of C(B) films was 10 min. The film thickness did not exceed 150 nm.

A WSe_2_ film was deposited by off-axis PLD on the surface of the substrate coated with a C(B) film. The substrate was rotated 90° and placed along the laser plume axis 2 cm away from the WSe_2_ target, which was manufactured by cold pressing of WSe_2_ powder [22]. During the WSe_2_ target ablation, the laser fluence was reduced to 4 J/cm^2^. The substrate temperature was 700 °С. WSe_2_ film deposition was performed in an Ar + 5% H_2_ mixture at a pressure of 15 Pa. The gas mixture was introduced into a chamber that had been evacuated to a residual gas pressure of 5 × 10^−4^ Pa or less. The deposition time of a WSe_2_ film of a thickness of ~200 nm was 20 min.

After the formation of WSe_2_ films, the sample was allowed to cool down to room temperature. Then it was rotated 90° for subsequent MoS*_x_* film deposition by PLD in the reaction gas. The Ar + 5% H_2_ gas mixture was pumped out with the help of a turbo-molecular pump, and H_2_S gas was introduced into the chamber until the pressure reached ~26 Pa. The Mo target ablation was carried out using 100 mJ pulses. The MoS*_x_* film deposition time was set at 6 min. The thickness of a deposited MoS*_x_* film on a smooth substrate did not exceed 20 nm. The choice of the Mo ablation conditions and the H_2_S pressure was motivated by the results of preliminary investigation of RPLD of MoS*_x_* films [38]. Under the chosen conditions of on-axis RPLD, the expected ratio was *х* = S/Mo~4.

### 2.2. Structural, Chemical, Electrical, Optical, and Photoelectrochemical Characterization Techniques

In this study, WSe_2_ films were produced by off-axis PLD for the first time, and thus they require further examination. Yet, C(B) and MoS*_x_* films obtained by on-axis (R)PLD have been studied extensively. We also discussed their structural and chemical properties in several publications. Therefore, in this article, we will focus on the information that will give a comprehensive picture of the components (layers) of the MoS*_x_*_~4_/WSe_2_/C(В) heterostructure.

The surface morphologies of the prepared films and heterostructures were examined by scanning electron microscopy (SEM, Tescan LYRA 3, Brno, Czech Republic). Using this microscope, the surface distribution of elements was studied by energy dispersive X-ray spectroscopy (EDS). The structure of the films was investigated by micro-Raman spectroscopy (MRS, Horiba, Kyoto, Japan), using a 632.8-nm (He-Ne) laser. The cross-section of the laser beam was <1 μm. To explore the structural features of WSe_2_ films obtained by off-axis PLD, the films were separated from the substrate and transferred onto metal grids to study by high-resolution transmission electron microscopy (HRTEM) and selected area electron diffraction (SAED) with the help of a JEM-2100, JEOL microscope (Toyo, Japan).

Band gaps (*E*_g_) in the prepared films were measured optically by processing absorption spectra. To this end, a Tauc plot was constructed to describe the dependence between (*αhν*)^1/r^ and (*hν*), where *α* is the absorption coefficient, *hν* is the photon energy, and *r* is a parameter that is taken to be 2 for indirect transitions. The optical absorption and transmission spectra were measured using an Agilent Technologies Cary Series UV-Vis-NIR spectrophotometer. Special samples were manufactured to explore the optical properties of WSe_2_ and MoS*_x_* films. In these samples, WSe_2_ and MoS*_x_* films were deposited on transparent sapphire substrates in the selected conditions.

The chemical states of WSe_2_ and MoS*_x_* films were studied by XPS. XPS spectra were obtained using a Theta Probe Thermo Fisher Scientific spectrometer with a monochromatic Al Kα X-ray source (*hν* = 1486.7 eV) and a 400 μm X-ray spot. The spectrometer energy scale was calibrated using Au4f_7/2_ core level lines located at a binding energy of 84.0 eV. The Advantage Data Spectrum Processing program was used for deconvolution of the experimental XPS spectra. The Shirley background is an approximation method that was used for determining the background under an XPS peak. The peaks were fitted by symmetric convolution of Gaussian and Lorentzian functions. The ratios of atomic concentrations of elements (*x* = S/Mo) were calculated considering the intensities of Mo 3d and S 2p peaks and the corresponding Scofield’s Relative Sensitivity Factor.

The thickness of quite thick MoS_x_ films (thickness is ≥100 nm) was measured by SEM. For this, the Si substrate with the deposited thick MoS*_x_* film was cleaved and the vertical cross section was investigated by SEM. These measurements made it possible to estimate the deposition rate of the MoS*_x_* films during reactive PLD. The deposition rate was used to determine the time for preparation of a very thin MoS*_x_* film (thickness is ~3 nm). The thickness of this thin film was then estimated from the results of XPS studies for MoS*_x_*/WSe_2_ heterostructure. The thickness of thin MoS*_x_* film was estimated as quite adequate if the XPS spectra of both films (MoS*_x_* and WSe_2_) could be detected at the same time.

The XPS measurements were used to determine the mutual arrangement of valence bands (VB) in the semiconductor heterostructures. The employed technique is widely used to study the band structure in heterojunction the formation of which can cause a change in the energy distribution of electrons [35,39,40]. The leading edge of the valence band spectrum was approximated by a linear function using the least-square fit of the leading edge of the VB spectra. The position of the valence band maximum (VBM) was determined as the intersection of the approximating linear function and the baseline. Determining the shift between the core levels of semiconductors in the heterojunction made it possible to calculate the valence band offset (VBO).

To calculate the VBO in a MoS *_x_*/WSe_2_ heterostructure, a series of measurements was performed. Firstly, the XPS spectra of the Mo3d and W4f core levels were measured along with the spectra of the valence bands of quite thick MoS*_x_* and WSe_2_ films. Secondly, the spectra of the Mo3d and W4f core levels were measured for a MoS *_x_*_~4_/WSe_2_ heterostructure, in which the thickness of the upper layer (MoS*_х_*) did not exceed 3 nm. Thirdly, the VBO value for heterojunctions was calculated based on the formula:

VBO = (E_Mo3d5/2_ − E_W4f7/2_)_interface_ + (E_W4f7/2_ − VBM_W_)_bulk_ − (E_Mo3d5/2_ − VBM_Mo_) _bulk,_

where *VBM_W_* and *VBM_Mo_* are the energies of the upper edge of the valence band for WSe_2_ and MoS*_х_*, respectively. ‘Interface’ stands for spectra for heterojunctions, and ‘bulk’ for the spectra of thicker films on C(B)/Al_2_O_3_ substrates.

The work function (φ) needed to withdraw an electron from a WSe_2_ film was calculated using the formula φ = *hν* − *E^CutOff^* + *E_F_*, where *E^CutOff^* is the secondary electron cutoff, *E*_F_ is the Fermi level if these magnitudes are considered on a kinetic energy scale. The Fermi level was determined based on an analysis of the energy spectrum of the valence band. To enable XPS investigation, the samples were created on a conducting C(B) film. This way, charge storage was prevented in the sample. The zero-value point of the binding energy scale corresponded to the Fermi level. In this case, the *E^CutOff^* value marked on the kinetic energy scale coincides with φ.

The electrical properties of C(B) films on sapphire substrates were studied by a four-contact method in the van der Pauw geometry; Hall-effect measurements were performed at room temperature. A magnetic field, varying from 0 to 1 T, was used for Hall-effect characterization. Metallic contacts to the sample with a circular mesa were formed from an InSn alloy, and the linearity of the volt–ampere characteristics of all contacts was monitored. During the measurements, the direction of the current was switched to eliminate the effects of thermoelectric power. The resistivity was calculated by averaging the values from all pairs of contacts.

To study the photoelectrocatalytical properties of MoS*_x_*/WSe_2_/C(В)/Al_2_O_3_ samples, we irradiated these samples with 100 W Xe lamps in an 0.5 M H_2_SO_4_ aqueous solution. The light intensity was 100 mW/cm^2^. A three-electrode configuration was used to determine the photo-assisted current in an electric circuit with modified cathodes. The polarization curves were measured using linear sweep voltammetry (LSV) with a change in the applied potential from −100 to 400 mV and a scan rate of 2 mV/s. When measuring LSV curves and the time evolution of the photocurrent, the light source was turned on and off. For chronoamperometry measurements, the potential of the photocathode was 0 V (relative to the reversible hydrogen electrode, RHE).

## 3. Results

### 3.1. On-Axis PLD of C(B) Films

**Figure 2:**
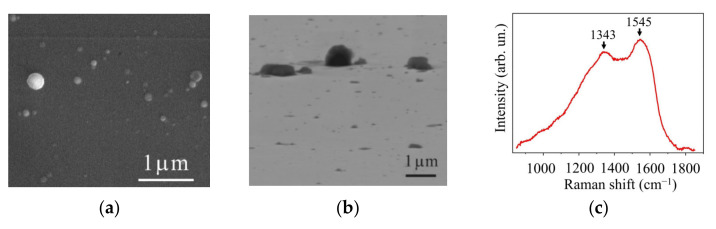
SEM images obtained at (**a**) normal and (**b**) 45° angles to the surface; (**c**) Raman spectrum of a С(В) film prepared on sapphire substrate by on-axis PLD in a vacuum.

Figure 2a,b show the morphology of a C(B) film formed on sapphire by traditional PLD. Detached rounded particles were observed on the smooth surface of C(B) films. The particle size ranged from 0.1 to 0.5 µm. This morphology is attributed to the deposition of B-rich particles [37]. The Raman spectrum of a C(B) film has two broad peaks at 1343 and 1545 cm^−1^ (Figure 2c), which correspond to the peaks marked D and G. The peaks are shifted to the lower wavenumber relative to the peaks associated with graphite (1360 cm^−1^ and 1580 cm^−1^ respectively). This shift meant that the films had a graphite-like local packing, which contained B atoms and some C atoms with sp^3^-bonding in sp^2^-matrix. A more detailed analysis of the Raman spectra of C(B) films obtained by PLD can be found in [37,41].

The C(B) films had a specific resistance of ~1.5 mΩ·cm and p-type conductivity. At room temperature, the carrier concentration and mobility were 4.4 × 10^19^ cm^−2^ and 180 cm^2^/V·s, respectively. The low resistance to the current flow in C(B) films enabled their use as a rear contact to the MoS*_x_*_~4_/WSe_2_ heterostructure, and p-type conductivity made it possible for holes formed upon illumination to move from the WSe_2_ film to the external electric circuit.

### 3.2. Off-Axis PLD of WSe_2_ Films

**Figure 3:**
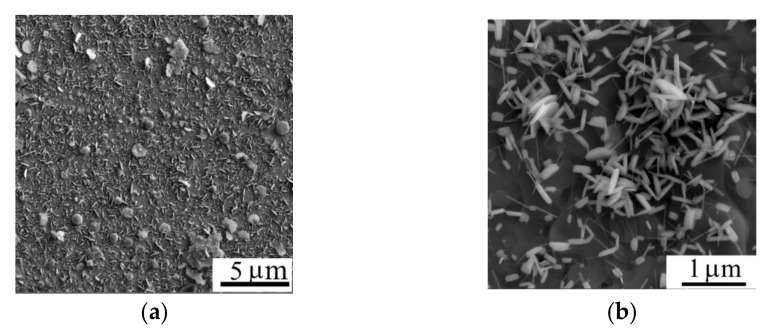
(**a**,**b**) SEM images of the surface of the WSe_2_ film (two magnifications) obtained by off-axis PLD on the surface of C(B)/Al_2_O_3_ sample.

Figure 3 shows the morphology of the WSe_2_ film obtained by off-axis PLD on the surface of C(B) film. The WSe_2_ film covers the surface of the C(B) film with a continuous layer and the WSe_2_ film had a nanocrystal structure consisted of petal-like crystals with random orientation relative to the film surface. The linear sizes of WSe_2_ crystals reached 1 µm, whereas the thickness of the nanopetals did not exceed 50 nm.

**Figure 4:**
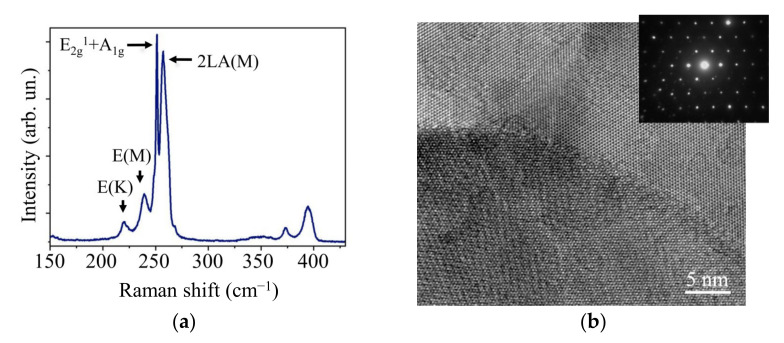
(**a**) Raman spectra and (**b**) HRTEM and SAED patterns of the WSe_2_ film obtained by off-axis PLD.

A structural investigation by MRS and TEM/MD techniques demonstrated that the WSe_2_ film had a crystal structure. An MRS spectrum (Figure 4a) only had peaks characteristic of the 2H-WSe_2_ phase. The peaks associated with the vibrational modes E_2g_^1^ and A_1g_ coincided because the shift between them was approximately 3 cm^−1^ [42,43]. The narrow half-height width of the peak (3 cm^−1^) points to the suitable quality of the crystal structure. A high-resolution TEM and SAED analysis of a single WSe_2_ petal showed that it consisted of several nanocrystals with a hexagonal lattice of the 2H-WSe_2_ phase (Figure 4b). The nanocrystal size was ~10 nm. Although the nanocrystals were oriented randomly relative to the *c*-axis, the basal plane of all the nanocrystals was parallel to the petal surface.

**Figure 5:**
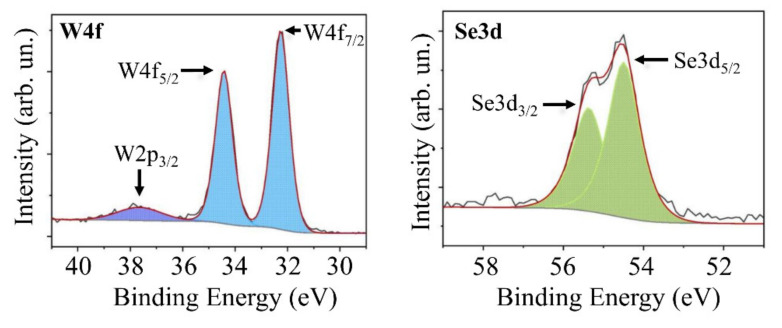
Core level XPS W4f and Se3d spectra of the WSe_2_ film obtained by off-axis PLD.

Figure 5 shows part of the XPS spectra for the surface of WSe_2_ film deposited on the surface of C(B) film. The W4f spectrum was well described by a doublet in which the W4f_7/2_ and W4f_5/2_ peaks had binding energies of 32.24 and 34.47 eV, respectively, which are characteristic of WSe_2_. The Se3d spectrum was described by a doublet whose Se3d_5/2_ and Se3d_3/2_ peaks were at 54.50 and 55.37 eV, respectively. The XPS spectra indicated effective chemical interaction between Se and W during off-axis PLD [13,43,44].

**Figure 6:**
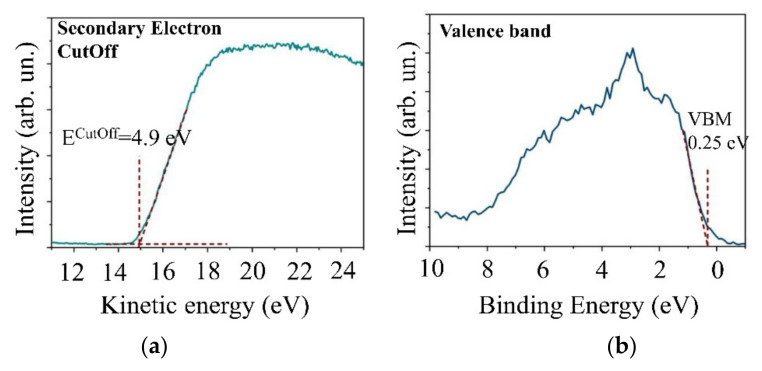
(**a**) XPS spectrum of secondary electrons cutoff and (**b**) evaluation of the valence band edge position for a WSe_2_ film obtained by off-axis PLD on the surface of C(B) film.

**Figure 7:**
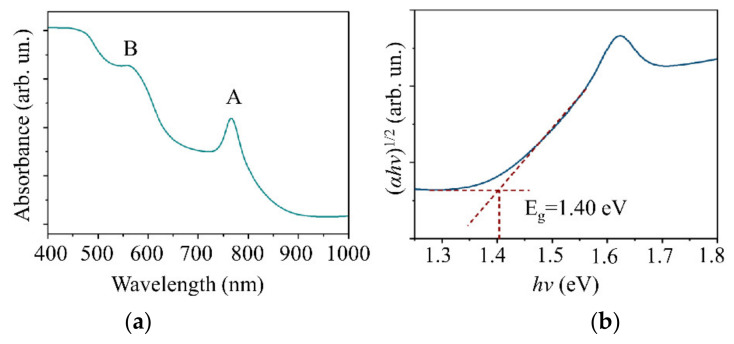
(**a**) Optical absorption spectra and (**b**) Tauc plots for the WSe_2_ film on the sapphire substrate. The A and B peaks are explained by excitonic absorption.

An analysis of the energy spectrum of secondary electrons and the valence band showed that the work function for WSe_2_ electrons was 4.9 eV (Figure 6a). The Fermi level was close to the bottom of the band gap 0.25 eV away from the upper edge of the valence band (Figure 6b). An investigation of the WSe_2_ film optical properties demonstrated that the film had an absorption spectrum characteristic of WSe_2_; the band gap width was 1.4 eV (Figure 7). A study of the band structure of the WSe_2_ film proved that it had p-type conductivity typical of this compound [45,46].

### 3.3. On-Axis Reactive PLD of MoS_x~4_ Film

**Figure 8:**
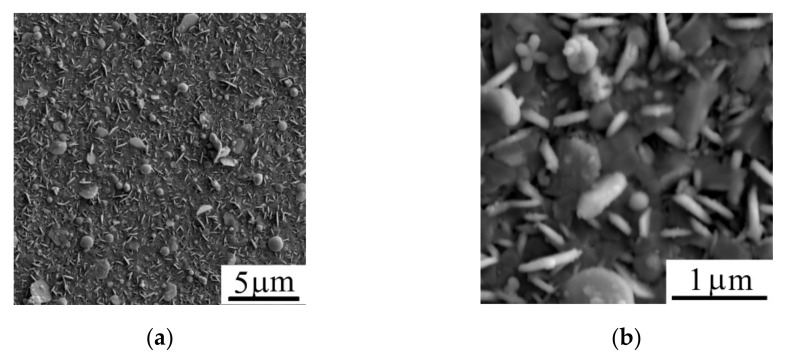
(**a**,**b**) SEM images (two magnifications) of the MoS*_x_*/WSe_2_/C(B)/Al_2_O_3_ sample.

**Figure 9:**
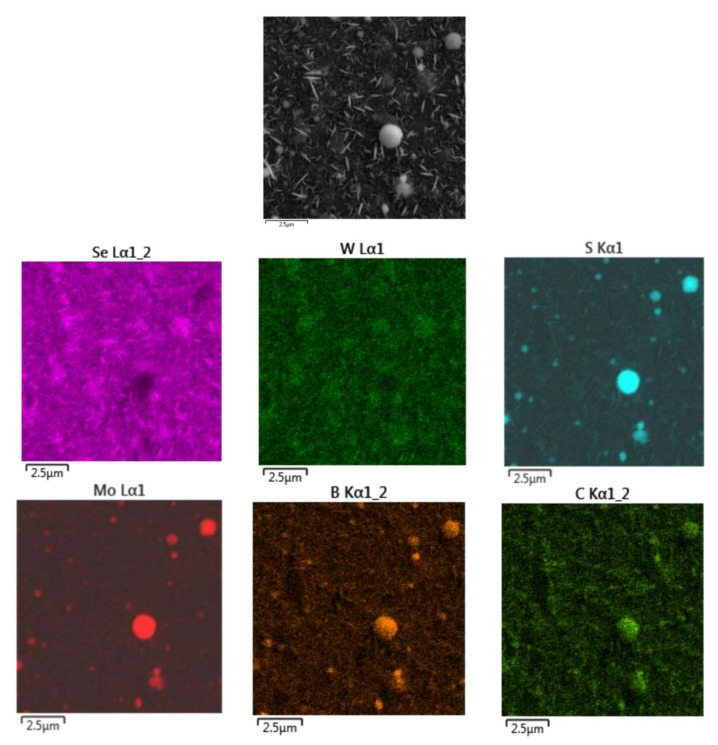
SEM image (top gray) and EDS maps (colored) of element distribution on the surface of the MoS_x_/WSe_2_/C(B)/Al_2_O_3_ sample. Intensity of different colors indicates where the corresponding elements (Se, W, S, Mo, B, and C) are most abundant. The presence of submicron rounded particles is explained by B-rich particle deposition during C(B) film formation.

Figure 8 shows an SEM image of the surface of WSe_2_/C(B)/Al_2_O_3_ sample after MoS*_x_* film deposition by on-axis reactive PLD. MoS*_x_* film deposition did not cause a substantial change in the morphology of the sample surface. The principal difference between SEM images of the WSe_2_ film before (Figure 3) and after MoS*_x_* film deposition (Figure 8) was that the sides of the WSe_2_ nanocrystal petals lost their sharpness when coated by a thin MoS*_х_* film, which is a porous structure. Mapping element distribution in the sample surface suggested that the MoS*_x_* film had a sufficiently homogeneous distribution over the sample surface (Figure 9). During PLD, the collision of Mo atoms with H_2_S molecules ensured their scattering at different angles. As a result, a MoS*_x_* film could be formed even on those WSe_2_ nanopetals that were oriented perpendicular to the surface of the substrate.

**Figure 10:**
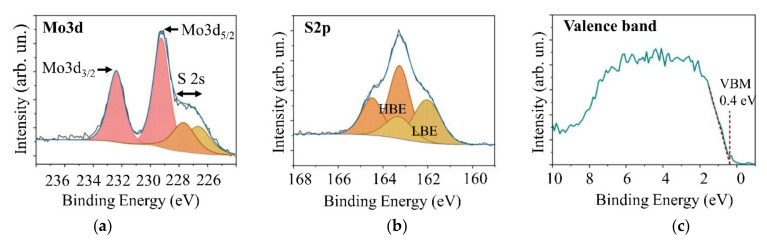
XPS spectra of (**a**,**b**) core level Mo3d and S2p and (**c**) the valence band of a relatively thick MoS*_x_* film obtained by on-axis RPLD.

Figure 10 shows the results of XPS investigation of a quite thick MoS*_x_* film obtained by on-axis RPLD. An analysis of the chemical state of elements showed (Figure 10a,b) that core level XPS Mo3d spectrum was well described by a doublet corresponding to the Mo^4+^ state. The bonding energy of the peak Mo3d_5/2_ was 229.24 V, accounted for by chemical bonds with S atoms [26,34]. Molybdenum oxides (Mo^6+^) or metallic Мо^0^ were not observed. The Mo3d_5/2_ peak partially overlapped with the S2s peak. The S2s peak consisted of singular peaks whose position correlated with that of doublets in the S2p spectrum. When analyzing the S2p peak, we used the traditional approach, i.e., we identified the states of sulfur with high and low binding energy (HBE and LBE, respectively) [34,35]. The LBE doublet was associated with single S^2-^ atoms (in MoS_2_-like clusters) and a terminal (S_2_^2−^)_tr_ ligand (in Mo_3_S_13_/Mo_3_S_12_ clusters). The doublet had S2p_3/2_ and S2p_1/2_ peaks with binding energies of 162.04 and 163.35 eV, respectively. The HBE doublet had S2p_3/2_ and S2p_1/2_ peaks, whose binding energies were 163.28 and 164.50 eV. This doublet is usually attributed to apical S^2-^ and bridging (S_2_^2−^)_br_ ligands in Mo_3_S_13_/Mo_3_S_12_ clusters. An XPS studies-based calculation of S/Mo atomic concentration ratios for this film confirmed that *x*~4.0. Measuring the valence band spectrum showed that the Fermi level was 0.4 eV away from the bottom of the band gap (Figure 10c).

**Figure 11:**
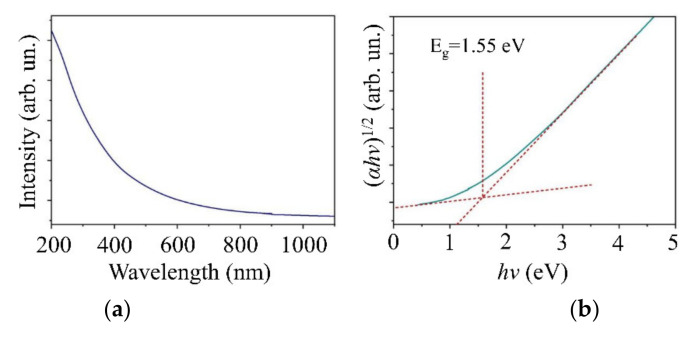
(**a**) Optical absorption spectra and (**b**) Tauc plots for the MoS*_x_*_~4_ film deposited on sapphire substrate.

Figure 11a shows the spectrum of optical absorption for the MoS*_x_*_~4_ film. Figure 11b demonstrates a Tauc plot calculated for that spectrum. The optical properties of MoS*_x_*_~4_ films are very similar to those of WSe_2_. This similarity sets a limit on the thickness of the MoS*_x_* film in a MoS*_x_*/WSe_2_ heterostructure since both films absorbed light most efficiently at wavelengths below 500 nm. The width of the band gap in a MoS*_x_* film was 1.55 eV. A Fermi level in the lower part of the band gap indicated p-type conductivity in the MoS*_x_*_~4_ film.

**Figure 12:**
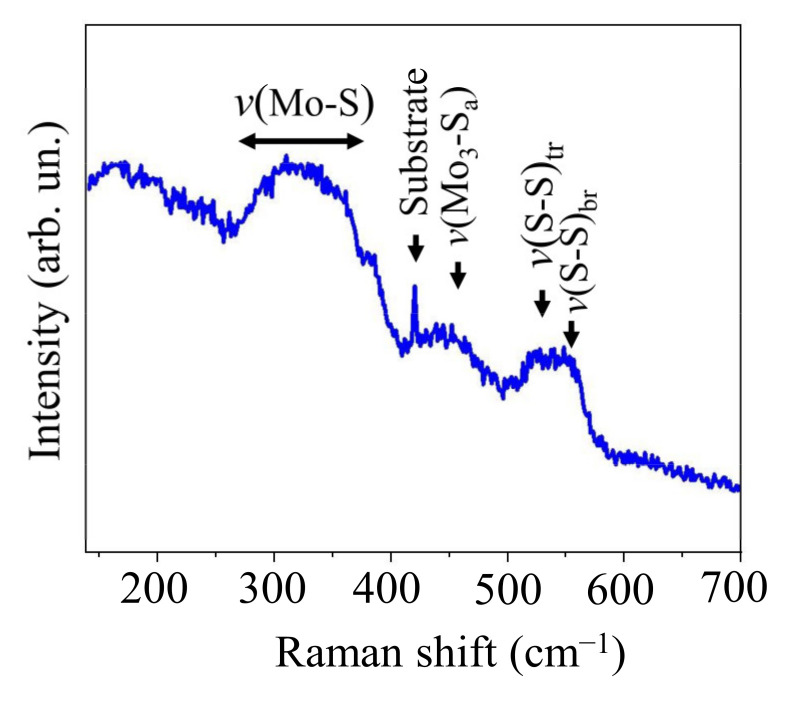
Raman spectra of MoS*_x_*_~4_ film obtained by on-axis RPLD on a sapphire substrate.

The local packing of atoms in the MoS*_x_*_~4_ film was investigated by MRS. It can be seen in Figure 12 that the Raman spectrum of the film consists of a set of broadened strips, whose position correlates well with that of the bands in the Raman spectrum of a catalytic molybdenum sulfide film obtained by chemical synthesis in a solution [13] and by reactive magnetron sputtering [47]. The spectrum had two clear broadened peaks at ~525 and ~550 cm^−1^, which were accounted for by the vibrational modes *ν*(S-S)_tr_ and ν(S-S)_br_ respectively in Mo_3_S_13_/Mo_3_S_12_ clusters. The peak at ~450 cm^−1^ is explained by the vibrations of apical S in Mo_3_S_13_ clusters. A broad band in the range 250–400 cm^−1^ is characteristic of an amorphous featureless structure of MoS*_x_*. Thus, the selected regime of on-axis RPLD made it possible to obtain thin layers of an amorphous molybdenum sulfide containing Mo_3_S_13_/Mo_3_S_12_ clusters on the surface of a nanostructured WSe_2_ film. The high electrocatalytic activity of such an amorphous molybdenum sulfide could contribute to a photo-assisted HER if the flux of nonequilibrium carriers (electrons) through the interface with WSe_2_ was sufficient.

### 3.4. Photoelectrocatalytic Properties of the MoS_x~4_/WSe_2_/C(B)/Al_2_O_3_ Cathode

**Figure 13:**
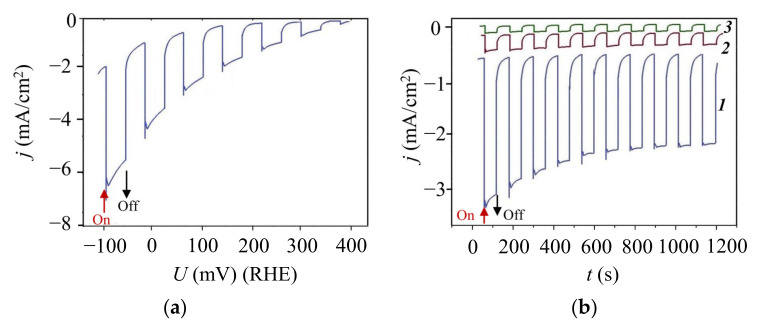
(**a**) Chopped LSV curve for the MoS*_x_*_~4_/WSe_2_/C(B)/Al_2_O_3_ photocathode in 0.5 M H_2_SO_4_ upon illumination; (**b**) Chopped photocurrent density versus time for MoS*_x_*_~4_/WSe_2_/C(B)/Al_2_O_3_ (curve ***1***), MoS_x~4_/C(B)/Al_2_O_3_ (curve ***2***) and WSe_2_/C(B)/Al_2_O_3_ (curve ***3***) photocathodes at 0 V (RHE) in 0.5 M H_2_SO_4_ upon illumination.

Figure 13 shows the results of an investigation of photoelectocatalytic properties of various heterostructure based on laser-deposited MoS*_x_*_~4_, WSe_2_, and C(B) films on a sapphire substrate. During photo-assisted HER, the MoS*_x_*_~4_/WSe_2_/C(B) materials combination had the most suitable properties (Figure 13a). A luminous flux caused the photo-current density to increase to ~3 мА/cm^2^ at a voltage of 0 V(RHE). The photocurrent magnitude was superimposed with the relatively high dark current raised due to transient effects [43]. The onset potential reached 400 mV (RHE). The heterojunction between MoS*_x_*_~4_ and WSe_2_ films was largely responsible for an efficient photo-assisted HER in this photocathode. Cathodes with a single semiconductive layer (MoS*_x_*_~4_ or WSe_2_) on the C(B) layer were associated with very low efficiency of photo-assisted HER (Figure 13b).

In the study of the temporal stability of the MoS*_x_*_~4_/WSe_2_/C(B)/Al_2_O_3_ photocathode, the current density was found to rapidly decrease by 20% in 20 min under chopped illumination. After a period of decline, the current density remained relatively stable for two hours. Longer tests of the temporal stability of this photocathode were not performed.

Table 1 contains collected data for comparison of the main parameters of metal chalcogenide-based photocathodes that characterize their performance in photo-assisted HER. It can be seen that the MoS*_x_*_~4_/WSe_2_/C(B)/Al_2_O_3_ photocathode created by pulsed laser deposition is not inferior in general in photo-assisted HER to the performance of photocathodes which were prepared by the methods of wet/dry chemical synthesis, exfoliation, spin coating, etc. Next, we will discuss the factors that should be overcome to enhance the photo-assisted HER efficiency of the MoS*_x_*_~4_/WSe_2_/C(B)/Al_2_O_3_ photocathode.

## 4. Discussion

**Figure 14:**
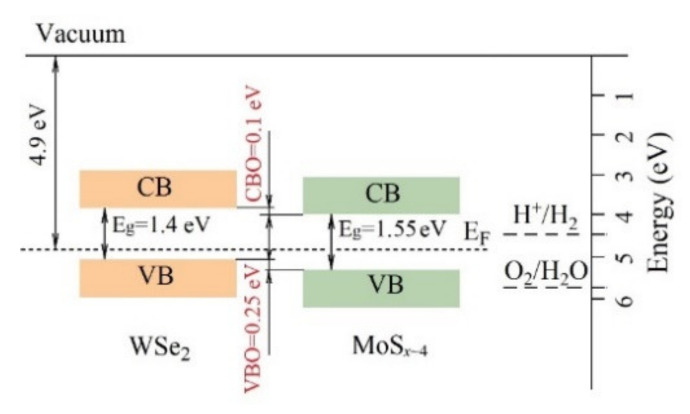
Band alignment diagram for the MoS *_x_*_~4_/WSe_2_ heterojunction system obtained by PLD.

XPS studies of MoS*_x_*_~4_ and WSe_2_ layers obtained by PLD showed that they had p-type conductivity. Such a combination of the electrophysical properties of contacting semiconductors creates a situation when the efficiency of photo-assisted HER processes largely depends on the structure of energy bands at the MoS*_x_*_~4_ /WSe_2_ interface. Figure 14 shows band alignment at the interface which was determined through a comprehensive study of the films by XPS and optical methods. The conductive band offset (CBO) value was calculated using the formula:

CBO = VBO *+ E_g_(*WSe_2_*)* − *E_g_(*MoS *_x_*_~4_*).*

The obtained СВО value equaled 0.1 eV. Thus, the band alignment was of type II, which is associated with the most efficient separation of photo-generated electron-hole pairs. In this case, electrons will move into the MoS*_x_* layer from the WSe_2_ and participate in the hydrogen evolution reaction, whilst holes will move from MoS*_x_* into the WSe_2_ layer. From WSe_2_, holes will migrate into the С(В) rear contact and further into the external electric circuit.

An additional study of MoS*_x_*_~4_/WSe_2_/C(B)/Al_2_O_3_ samples by electrochemical impedance spectroscopy (EIS) demonstrated that the С(В) contact layer did not ensure a sufficiently low resistance to the flow of current. The value of equivalent series resistance (*R*_s_), which was extracted from EIS data, achieved 30 Ω. When a glassy carbon conducting substrate was used to create a MoS*_x_*_~4_/WSe_2_/GC photocathode, *R*_s_ did not exceed 4 Ω, and the density of the photo-assisted HER current increased. Therefore, to increase the efficiency of a photo-assisted HER when using a MoS *_x_*_~4_/WSe_2_ heterojunction system, it is recommended to choose a rear contact with an electrical resistance lower than that of the C(B) film. Further work may focus on the effect of the B concentration on the electrical properties of such films.

The analysis of the optical characteristics of the MoS*_x_*_~4_ and WSe_2_ films showed that these films absorb light rather efficiently. These nanomaterials are potentially active catalysts for the hydrogen evolution reaction. However, these factors did not provide effective photo-assisted HER in the MoS*_x_*_~4_/C(B)/Al_2_O_3_ and WSe_2_/C(B)/Al_2_O_3_ samples. Additional experiments with thicker MoS*_x_*_~4_ and WSe_2_ films did not reveal significant changes in the efficiency of photo-assisted HER. This indicated that after the generation of electron-hole pairs under a light flux, electrons and holes could rapidly recombine in the bulk of the films. The formation of a heterojunction turned out to be the most important factor contributing to an increase in the photocurrent. At the interface of the MoS*_x_*_~4_ and WSe_2_ films, not only the processes of separation of nonequilibrium electrons and holes due to the specificity of the energy bands alignment could occur, but also recombination processes can be expected. The recombination processes will facilitate to photo-assisted HER if electrons from WSe_2_ and holes from MoS*_x_*_~4_ actively participated in the recombination process (Z-schema) [35]. However, one cannot exclude the recombination at this interface of electrons and holes generated by the light flux in the WSe_2_ film. In addition, the small size of the crystalline domains in the WSe_2_ film and their random orientation resulted in a high density of edge states. This should lead to a decrease in the efficiency of charge separation since such edge states serve as recombination centers [13]. Insufficiently large values of CBO and VBO for the MoS*_x_*_~4_/WSe_2_ heterojunction could also be the reason limiting the efficiency of photo-assisted HER in our samples.

**Figure 15:**
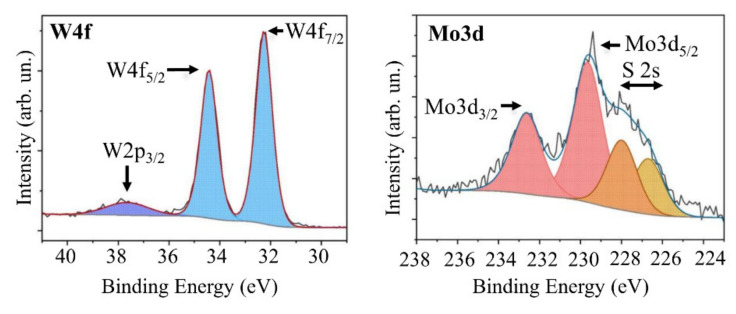
XPS W4f and Mo3d (overlapped with S2s) spectra for a very thin MoS*_x_* film obtained by on-axis reactive PLD on the surface of WSe_2_ layer.

Another factor that could reduce the efficiency of a photo-assisted HER with a MoS*_x_*_~4_/WSe_2_/C(B)/Al_2_O_3_ photocathode is modification of the MoS*_x_*_~4_/WSe_2_ interface under the influence of hydrogen sulfide activated by laser-induced plasma. Figure 15 shows W4f and Mo3d XPS spectra measured for a very thin MoS*_x_* film formed by on-axis RPLD on the surface of the WSe_2_ layer. A comparison of these spectra with those of pristine WSe_2_ and MoS*_x_*_~4_ (Figure 5 and Figure 10) demonstrated that the chemical state of W has practically not changed after the deposition of MoS*_x_* film. The Mo3d spectrum shifted by 0.37 eV towards greater bonding energies, whilst the S2s spectrum increased in intensity. These changes indicated that, at the initial stage of the MoS*_x_* film growth, sulfur could be effectively deposited on the WSe_2_ as a result of H_2_S molecules interacting with the WSe_2_ surface. The plasma that formed in H_2_S during the ablation of the Мо target could activate the process. The introduction of S atoms into the WSe_2_ crystal lattice can distort the latter and thus cause the formation of new energy levels in the WSe_2_ band gap. At the same time, energy bands will bend in the contact area. Band bending may cause a bonding energy shift for the Mo3d_5/2_ peak and increase the width at the half maximum of the peak from 1.4 to 1.8 eV. At these energy levels, effective recombination of electrons and holes formed upon illumination may occur.

To change the conditions under which the MoS*_x_*_~4_/WSe_2_ interface is formed, one can employ a different technique for the deposition of a molybdenum sulfide film—one that prevents the influence of plasma-activated H_2_S gas. Fominski et al. [55] and Giuffredi et al. [32] demonstrate that the pulsed laser ablation of a MoS_2_ target in a buffer gas enables the formation of MoS*_x_* films with an increased concentration of sulfur (*x* ≥ 3). These films have an extremely high electrocatalytic HER activity. The area of MoS*_x_* film deposition (i.e., WSe_2_ nanopetals) can be oriented randomly to the axis of the plume expansion [56]. If this technique for MoS*_x_* film deposition is applied, the deposition of Mo and S atom flux on the interface with the WSe_2_ film occurs almost simultaneously. This contributes to the formation of Mo‒S chemical bonds in the growing film. The energy of atoms deposited during the ablation of the MoS_2_ target in the on-axis PLD configuration is much lower than during the ablation of metallic Mo in the on-axis RPLD configuration. As a rule, the ablation of metals occurs in the conditions of effective laser plume ionization under the influence of more powerful laser pulses. This factor can also impact chemical processes at the MoS*_x_*/WSe_2_ interface.

The regulation of the MoS*_x_*/WSe_2_ interface formation is not the only factor that affects the efficiency of photo-assisted HER. Another one is the texture of the WSe_2_ layer [10,13]. WSe_2_ petals sitting along the substrate surface minimize the impact of edge states on the recombination on nonequilibrium carriers. Yet, the orthogonal orientation of the petals increases the area of the surface involved in catalysis. The negative effect of edge states can be reduced through their passivation by a MoS*_x_* catalyst. We carried out additional studies to obtain WSe_2_ by off-axis PLD at varying buffer gas pressures. This factor did not have a marked effect on the texture of WSe_2_ films. When this WSe_2_ formation technique is used, other parameters of off-axis PLD may vary as well. These are laser fluence, the laser plume incidence angle, deposition temperature, etc. Co-deposition with some metals (for example, Pd [13]) will also affect the growth of WSe_2_ films. The optimization of regimes for obtaining WSe_2_ films with a targeted structure by laser-based methods is a central condition for creating HER photocatalysts with suitable characteristics. Achieving the latter requires further research into the MoS*_x_*/WSe_2_ heterojunction system.

## 5. Conclusions

Using different PLD configurations makes it possible to fully form a HER catalyst (in one production vessel) on a dielectric substrate (sapphire). A robust rear contact (conducting layer) was obtained using B-doped amorphous carbon by traditional on-axis PLD. A nanostructured WSe_2_ layer was grown on the C(B) contact layer. The WSe_2_ layer consisted of differently oriented nano-petals, which had a nearly perfect 2H-WSe_2_ crystal lattice. To obtain a WSe_2_ layer, off-axis PLD was performed in a buffer gas. A catalytic MoS*_x_*_~4_ layer was created on the surface of WSe_2_ petals by on-axis reactive PLD from Mo target in H_2_S gas. The temperature of functional layer formation for a MoS*_x_*_~4_/WSe_2_/C(B)/Al_2_O_3_ photocathode ranged between 22–700 °С.

The MoS*_x_*_~4_/WSe_2_/C(B)/Al_2_O_3_ photocathode obtained by laser-based processes has the following characteristics as regards HER in 0.5M H_2_SO_4_ acid solution during light irradiation with an intensity of 100 mW/cm^2^: the current density at 0 V (RHE) is ~3 мА/cm^2^; the onset potential reaches 400 mV (RHE). Given that these photocathodes are made from relatively cheap materials commonly found in nature, these are suitable characteristics. The performance of MoS*_x_*/WSe_2_ heterojunction system for photo-assisted water splitting for hydrogen production can be substantially increased by enhancing the composition of the photocatalyst (i.e., employing a different rear contact) and optimizing PLD regimes for creating functional semiconductor layers.

## Figures and Tables

**Table 1:** Comparison of photo-assisted HER performances for metal chalcogenide-based photocathodes with heterojunction structure.

Hetero- Structures	Rear Contact/ Support	Preparation Methods	*U*_onset_, mV (RHE)	Photocurrent at *U* = 0, mA/cm^2^	Light Intensity, mW/cm^2^	Ref.
WSe_2_(Pt)	TiN:O/ SiO_2_/Si	aSLcS process *^1^	~500	≤1	100	[48]
(NH4)_2_Mo_3_S_13_/WSe_2_	TiN:O/ quarts glass	Spin coating/ aSLcS	~250	5.6	100	[13]
MoS*_x_*O*_y_* /2D-WSe_2_	F:SnO_2_/ glass	SDCI *^2^/ drop casting	~300	2.0	100	[49]
Mo_x_S_y_/WSe2	rGO/F:SnO_2_/ glass	Drop casting/ successive dip coating	~0.2	~3–4	100	[50]
WSe_2_-PANI (Polyaniline) nanohybrid		Vapor transport technique	280	~20	30	[51]
WSe_2_(Pt-Cu)	F:SnO_2_/glass	Exfoliation/ spin-coating	~350	~4	100	[52]
Pt/(NH_4_)_2_oS_4_/WSe_2_	TiN:O/glass	aSLcS/spin coating	~200	~5	100	[14]
MoS_2_/WSe_2_	F:SnO_2_/glass	mechanical exfoliation/chemical vapor deposition	800 (SCE)	0.4	100	[53]
p-WSe_2_/FePt	Metallic tungsten substrate	Chemical vapor transport	200	4	100	[54]
MoS_4_/WSe_2_	C(B)/Al_2_O_3_	RPLD/PLD	400	3	100	This work

*^1^ the amorphous solid–liquid–crystalline solid process with Pd promoter. *^2^ selective dip coating impregnation.

## Data Availability

Not applicable.

## References

[1] Wang F., Shifa T.A., Zhan X., Huang Y., Liu K., Cheng Z., Jiang C., He J. (2015). Recent advances in transition-metal dichalcogenide based nanomaterials for water splitting. Nanoscale.

[2] Zhang Q., Wang W., Zhang J., Zhu X., Zhang Q., Zhang Y., Ren Z., Song S., Wang J., Ying Z. (2018). Highly Efficient Photocatalytic Hydrogen Evolution by ReS_2_ via a Two-Electron Catalytic Reaction. Adv. Mater..

[3] Andoshe D., Jeon J., Kim S., Jang H. (2015). Two-Dimensional Transition Metal Dichalcogenide Nanomaterials for Solar Water Splitting. Electron. Mater. Lett..

[4] Wang Q., Kalantar-Zadeh K., Kis A., Coleman J., Strano M. (2012). Electronics and optoelectronics of two-dimensional transition metal dichalcogenides. Nat. Nanotechnol..

[5] Lu Q., Yu Y., Ma Q., Chen B., Zhang H. (2016). 2D Transition-Metal-Dichalcogenide-Nanosheet-Based Composites for Photocatalytic and Electrocatalytic Hydrogen Evolution Reactions. Adv. Mater..

[6] Huang X., Zeng Z., Zhang H. (2013). Metal dichalcogenide nanosheets: Preparation, properties and applications. Chem. Soc. Rev..

[7] Laursen A., Kegnæs S., Dahl S., Chorkendorff I. (2012). Molybdenum sulfides-efficient and viable materials for electro- and photoelectrocatalytic hydrogen evolution. Energy Environ. Sci..

[8] Luo Z., Wang T., Gong J. (2018). Single-crystal silicon-based electrodes for unbiased solar water splitting: Current status and prospects. Chem. Soc. Rev..

[9] Lin H., Li S., Yang G., Kai Zhang K., Tang D., Su Y., Li Y., Luo S., Chang K., Ye J. (2020). In Situ Assembly of MoS*_x_* Thin-Film through Self-Reduction on p-Si for Drastic Enhancement of Photoelectrochemical Hydrogen Evolution. Adv. Funct. Mater..

[10] McKone J., Adam P., Pieterick A., Gray H., Nathan S., Lewis N. (2013). Hydrogen Evolution from Pt/Ru-Coated p-Type WSe_2_ Photocathodes. J. Am. Chem. Soc..

[11] Li C., Cao Q., Wang F., Xiao Y., Li Y., Delaunay J.-J., Zhu H. (2018). Engineering graphene and TMDs based van der Waals heterostructures for photovoltaic and photoelectrochemical solar energy conversion. Chem. Soc. Rev..

[12] Zhong S., Xi Y., Wu S., Liu Q., Zhao L., Bai S. (2020). Hybrid cocatalysts in semiconductor based photocatalysis and photoelectrocatalysis. J. Mater. Chem. A.

[13] Bozheyev F., Xi F., Plate P., Dittrich T., Fiechter S., Ellmer K. (2019). Efficient charge transfer at a homogeneously distributed (NH_4_)_2_Mo_3_S_13_/WSe_2_ heterojunction for solar hydrogen evolution. J. Mater. Chem. A.

[14] Bozheyev F., Xi F., Ahmet I., Hohn C., Ellmer K. (2020). Evaluation of Pt, Rh, SnO_2_, (NH_4_)_2_Mo_3_S_13_, BaSO_4_ protection coatings on WSe_2_ photocathodes for solar hydrogen evolution. Int. J. Hydrog. Energy.

[15] Zhang W., Chiu M.-H., Chen C.-H., Chen W., Li L.-J., Thye A., Wee S. (2014). Role of Metal Contacts in High Performance Phototransistors Based on WSe_2_ Monolayers. Am. Chem. Soc..

[16] Chaudhary S., Umar A., Mehta S.K. (2016). Selenium nanomaterials: An overview of recent developments in synthesis, properties and potential applications. Prog. Mater. Sci..

[17] Li H., Zou J., Xie S., Leng X., Gao D., Yang H., Mao X. (2017). WSe_2_ nanofilms grown on graphite as efficient electrodes for hydrogen evolution reactions. J. Alloys Compd..

[18] Fominski V.Y., Grigoriev S.N., Romanov R.I., Volosova M.A., Grunin A.I., Teterina G.D. (2016). The Formation of a Hybrid Structure from Tungsten Selenide and Oxide Plates for a Hydrogen-Evolution Electrocatalyst. Tech. Phys. Lett..

[19] Zheng Z., Zhang T., Yao J., Zhang Y., Xu J., Yang G. (2016). Flexible, transparent and ultra-broadband photodetector based on large-area WSe_2_ film for wearable devices. Nanotechnology.

[20] Grigoriev S.N., Fominski V.Y., Nevolin V.N., Romanov R.I., Volosova M.A., Irzhak A.V. (2016). Formation of Thin Catalytic WSe*_x_* Layer on Graphite Electrodes for Activation of Hydrogen Evolution Reaction in Aqueous Acid. Inorg. Mater. Appl. Res..

[21] Seo S., Choi H., Kim S.-Y., Lee J., Kim K., Yoon S., Lee B., Lee S. (2018). Growth of Centimeter-Scale Monolayer and Few-Layer WSe_2_ Thin Films on SiO_2_/Si Substrate via Pulsed Laser Deposition. Adv. Mater. Interfaces.

[22] Grigoriev S.N., Fominski V.Y., Romanov R.I., Gnedovets A.G., Volosova M.A. (2013). Shadow masked pulsed laser deposition of WSe*_x_* films: Experiment and modeling. Appl. Surf. Sci..

[23] Fominski V.Y., Grigoriev S.N., Gnedovets A.G., Romanov R.I., Volosova M.A. (2013). Experimental study and modelling of laser plasma ion implantation for WSe*_x_*/^57^Fe interface modification. Appl. Surf. Sci..

[24] Fominski V.Y., Grigoriev S.N., Romanov R.I., Volosova M.A., Demin M.V. (2015). Chemical composition, structure and light reflectance of W-Se and W-Se-C films prepared by pulsed laser deposition in rare and reactive buffer gases. Vacuum.

[25] Fominski V.Y., Grigoriev S.N., Gnedovets A.G., Romanov R.I. (2013). On the Mechanism of Encapsulated Particle Formation during Pulsed Laser Deposition of WSe_x_ Thin-Film Coatings. Tech. Phys. Let..

[26] Fominski V.Y., Markeev A.M., Nevolin V.N., Prokopenko V.B., Vrublevski A.R. (1994). Pulsed laser deposition of MoS_x_ films in a buffer gas atmosphere. Thin Solid Films.

[27] Fominski V.Y., Nevolin V.N., Romanov R.I., Smurov I. (2001). Ion-assisted deposition of MoS*_x_* films from laser-generated plume under pulsed electric field. J. Appl. Phys..

[28] Fominski V., Demin M., Fominski D., Romanov R., Goikhman A., Maksimova K. (2020). Comparative study of the structure, composition, and electrocatalytic performance of hydrogen evolution in MoS*_x_*_~2+δ_/Mo and MoS*_x~_*_3+δ_ films obtained by pulsed laser deposition. Nanomaterials.

[29] Li B., Jiang L., Li X., Cheng Z., Ran P., Zuo P., Qu L., Zhang J., Lu Y. (2019). Controllable Synthesis of Nanosized Amorphous MoS_x_ Using Temporally Shaped Femtosecond Laser for Highly Efficient Electrochemical Hydrogen Production. Adv. Funct. Mater..

[30] Li Y., Yu Y., Huang Y., Nielsen R., William A., Goddard W.A., Li Y., Cao L. (2015). Engineering the Composition and Crystallinity of Molybdenum Sulfide for High-Performance Electrocatalytic Hydrogen Evolution. ACS Catal..

[31] Ding R., Wang M., Wang X., Wang H., Wang L., Mu Y., Lv B. (2019). N-Doped amorphous MoS*_x_* for the hydrogen evolution reaction. Nanoscale.

[32] Giuffredi G., Mezzetti A., Perego A., Mazzolini P., Prato M., Fumagalli F., Lin Y.-C., Liu C., Ivanov I., Belianinov A. (2020). Non-Equilibrium Synthesis of Highly Active Nanostructured, Oxygen-Incorporated Amorphous Molybdenum Sulfide HER Electrocatalyst. Small.

[33] Wang R., Sun P., Wang H., Wang X. (2017). Pulsed laser deposition of amorphous molybdenum disulfide films for efficient hydrogen evolution reaction. Electrochim. Acta.

[34] Fominski V.Y., Romanov R.I., Fominski D.V., Shelyakov A.V. (2017). Regulated growth of quasi-amorphous MoS*_x_* thin-film hydrogen evolution catalysts by pulsed laser deposition of Mo in reactive H_2_S gas. Thin Solid Films.

[35] Fominski V., Romanov R., Fominski D., Soloviev A., Rubinkovskaya O., Demin M., Maksimova K., Shvets P., Goikhman A. (2020). Performance and Mechanism of Photoelectrocatalytic Activity of MoS*_x_*/WO_3_ Heterostructures Obtained by Reactive Pulsed Laser Deposition for Water Splitting. Nanomaterials.

[36] Yang X., Liu W., Bastiani M., Allen T., Kang J., Xu H., Aydin E., Xu L., Bi Q., Dang H. (2019). Dual-Function Electron-Conductive, Hole-Blocking Titanium Nitride Contacts for Efficient Silicon Solar Cells. Joule.

[37] Fominski V.Y., Romanov R.I., Vasil’evskii I.S., Safonov D.A., Soloviev A.A., Zinin P.V., Bulatov K.M., Filonenko V.P. (2019). Structural, electrical and mechanical properties of ВС*_х_* films prepared by pulsed laser deposition from mixed and dual boron-diamond/graphite targets. Diam. Relat. Mater..

[38] Fominski V., Demin M., Nevolin V., Fominski D., Romanov R., Gritskevich M., Smirnov N. (2020). Reactive Pulsed Laser Deposition of Clustered-Type MoS*_x_* (*x*~2, 3, and 4) Films and Their Solid Lubricant Properties at Low Temperature. Nanomaterials.

[39] Chiu M., Zhang C., Shiu H., Chuu C., Chen C., Chang C.S., Chen C., Chou M., Shih C., Li L. (2015). Determination of band alignment in the single-layer MoS_2_/WSe_2_ heterojunction. Nat. Commun..

[40] Xing S., Zhao G., Wang J., Xu Y., Ma Z., Li X., Yang W., Liu G., Yang J. (2020). Band alignment of wo-dimensional h-BN/MoS_2_ van der Waals heterojunction measured by X-ray photoelectron spectroscopy. J. Alloys Compd..

[41] Fominski V.Y., Romanov R.I., Vasil’evskii I.S., Safonov D.A., Soloviev A.A., Ivanov A.A., Zinin P.V., Krasnoborodko S.Y., Vysokikh Y.E., Filonenko V.P. (2021). Pulsed laser modification of layered B-C and mixed BC*_x_* films on sapphire substrate. Diam. Relat. Mater..

[42] Luo X., Zhao Y., Zhang J., Toh M., Kloc C., Xiong Q., Quek S.Y. (2013). Effect of lower symmetry and dimensionality on Raman spectra in two-dimensional WSe_2_. Phys. Rev. B.

[43] Yu X., Prévot M.S., Guijarro N., Sivula K. (2015). Self-assembled 2D WSe2 thin films for photoelectrochemical hydrogen production. Nat. Commun..

[44] Boscher N.D., Carmalt C.J., Parkin I.P. (2006). Atmospheric pressure chemical vapor deposition of WSe_2_ thin films on glass-highly hydrophobic sticky surfaces. J. Mater. Chem..

[45] Lee C., Lee G., Van der Zande A.M., Chen W., Li Y., Han M., Cui X., Arefe G., Nuckolls C., Heinz T.F. (2014). Atomically thin p–n junctions with van der Waals heterointerfaces. Nat. Nanotechnol..

[46] Doan M., Jin Y., Adhikari S., Lee S., Zhao J., Lim S.C., Lee Y.H. (2017). Charge Transport in MoS2/WSe2 van der Waals Heterostructure with Tunable Inversion Layer. ACS Nano.

[47] Xi F., Bogdanoff P., Harbauer K., Plate P., Höhn C., Rappich J., Wang B., Han X., Van de Krol R., Fiechter S. (2019). Structural transformation identification of sputtered amorphous MoSx as efficient hydrogen evolving catalyst during electrochemical activation. ACS Catal..

[48] Bozheyev F., Rengacharid М., Berglunde S., Abou-Rase D., Ellmere K. (2019). Passivation of recombination active PdSe_x_ centers in (001)-textured photoactive WSe_2_ films. Mat. Sci. Semicon. Proc..

[49] Barbosa J.B., Taberna P.L., Bourdon V., Gerber I.C., Poteau R., Balocchi A., Marie X., Esvan J., Puech P., Barnabé A. (2020). Mo thio and oxo-thio molecular complexes film as self-healing catalyst for photocatalytic hydrogen evolution on 2D materials. Appl. Catal. B.

[50] Taberna P.L., Barbosa J.B., Balocchi A., Gerber K.U., Barnabe A., Marie X., Chane-Ching J.Y. (2021). Patch-like, Two Dimensional WSe_2_-Based Hetero-structures Activated by a Healing Catalyst for H_2_ Photocatalytic Generation. Chem. Eng. J..

[51] Kannichankandy D., Pataniya P.M., Sumesh C.K., Solanki G.K., Pathak V.M. (2021). WSe_2_-PANI nanohybrid structure as efficient electrocatalyst for photo-enhanced hydrogen evolution reaction. J. Alloys Compd..

[52] Yu X., Guijarro N., Johnson M., Sivula K. (2018). Defect mitigation of Solution-Processed 2D WSe_2_ Nano-flakes for Solar-to Hydrogen Conversion. Nano Lett..

[53] Si K., Ma J., Lu C., Zhou Y., He C., DanYang D., Wang X., Xu X. (2020). A two-dimensional MoS_2_/WSe_2_ van der Waals heterostructure for enhanced photoelectric performance. Appl. Surf. Sci..

[54] Zheng X., Zhang G., Xu X., Liu L., Zhang J., Xu Q. (2019). Synergistic effect of mechanical strain and interfacial-chemical interaction for stable 1T-WSe_2_ by carbon nanotube and cobalt. Appl. Surf. Sci..

[55] Fominski V.Y., Romanov R.I., Fominski D.V., Dzhumaev P.S., Troyan I.A. (2018). Normal and grazing incidence pulsed laser deposition of nanostructured MoS*_x_* hydrogen evolution catalysts from a MoS_2_ target. Opt. Laser Technol..

[56] Nevolin V.N., Fominski D.V., Romanov R.I., Esin M.I., Fominski V.Y., Kartsev P.F. (2019). Selection of pulsed laser deposition conditions for preparation of perfect thin-film MoS*_x_* hydrogen evolution catalysts. J. Phys. Conf. Ser..

